# Influence of calcium ion-modified implant surfaces in protein adsorption and implant integration

**DOI:** 10.1186/s40729-021-00314-1

**Published:** 2021-04-21

**Authors:** Eduardo Anitua, Andreia Cerqueira, Francisco Romero-Gavilán, Iñaki García-Arnáez, Cristina Martinez-Ramos, Seda Ozturan, Mikel Azkargorta, Félix Elortza, Mariló Gurruchaga, Isabel Goñi, Julio Suay, Ricardo Tejero

**Affiliations:** 1 University Institute of Regenerative Medicine and Oral Implantology (UIRMI), University of the Basque Country (UPV-EHU), C/ Jacinto Quincoces, 39, 01007 Vitoria, Spain; 2 Department of Industrial Systems Engineering and Design, Universitat Jaume I, Av. Vicent Sos Baynat s/n, 12071 Castellón de la Plana, Spain; 3 Faculty of Chemical Sciences, University of the Basque Country (UPV-EHU), P.M. de Lardizábal, 3, 20018 San Sebastián, Spain; 4 Center for Biomaterials and Tissue Engineering, Universitat Politècnica de Valencia, Camino de Vera, s/n, 46022 Valencia, Spain; 5 Department of Periodontology, Faculty of Dentistry, Istambul Medeniyet University, Istanbul, Turkey; 6 Proteomics Platform, CIC bioGUNE, CIBERehd, ProteoRed-ISCIII, Bizkaia Science and Technology Park, 48160 Derio, Spain

**Keywords:** Titanium implants, Osseointegration, Blood coagulation, Implant surface design, Protein adsorption

## Abstract

**Background:**

Calcium (Ca) is a well-known element in bone metabolism and blood coagulation. Here, we investigate the link between the protein adsorption pattern and the in vivo responses of surfaces modified with calcium ions (Ca-ion) as compared to standard titanium implant surfaces (control). We used LC–MS/MS to identify the proteins adhered to the surfaces after incubation with human serum and performed bilateral surgeries in the medial section of the femoral condyles of 18 New Zealand white rabbits to test osseointegration at 2 and 8 weeks post-implantation (*n*=9).

**Results:**

Ca-ion surfaces adsorbed 181.42 times more FA10 and 3.85 times less FA12 (*p*<0.001), which are factors of the common and the intrinsic coagulation pathways respectively. We also detected differences in A1AT, PLMN, FA12, KNG1, HEP2, LYSC, PIP, SAMP, VTNC, SAA4, and CFAH (*p*<0.01). At 2 and 8 weeks post-implantation, the mean bone implant contact (BIC) with Ca-ion surfaces was respectively 1.52 and 1.25 times higher, and the mean bone volume density (BVD) was respectively 1.35 and 1.13 times higher. Differences were statistically significant for BIC at 2 and 8 weeks and for BVD at 2 weeks (*p*<0.05).

**Conclusions:**

The strong thrombogenic protein adsorption pattern at Ca-ion surfaces correlated with significantly higher levels of implant osseointegration. More effective implant surfaces combined with smaller implants enable less invasive surgeries, shorter healing times, and overall lower intervention costs, especially in cases of low quantity or quality of bone.

**Supplementary Information:**

The online version contains supplementary material available at 10.1186/s40729-021-00314-1.

## Background

Titanium (Ti) is the preferred material for biomedical applications because of its balance of mechanical properties, corrosion resistance, biocompatibility, and osseointegration [1]. Implant surface characteristics play a crucial role in the physiological acceptance of implanted materials. Many surface modifications have been proposed aiming at improving implant osseointegration. These modifications gravitate mainly around roughness and/or oxide composition and, more recently, incorporate bioactive agents to the surfaces [2]. Research in this field has led to the development of implant surfaces modified with specific molecules and bioinorganic ions that enhance the intrinsic osteogenic capacity of Ti, leading to specific physical and biochemical responses in the bone tissue around the implant [3, 4].

The first biological process that takes place upon implant placement is blood protein adsorption and the formation of a blood clot onto the biomaterial surface. These processes are modulated by material’s physicochemical properties such as chemical composition, surface morphology, and charge [5]. Advances in the knowledge of the molecular and biochemical pathways involved in bone regeneration show the importance of elements such as calcium, strontium, magnesium, or zinc [6, 7]. Calcium (Ca) ions, for example, promote and accelerate blood coagulation leading to the formation of the prothrombinase complex, which converts prothrombin into thrombin and, thereby, fibrinogen into fibrin [8–10]. The characteristics of the fibrin architecture of the blood clot are relevant to give shape and function to the forming implant-surface scaffold that mediates the adhesion, proliferation, and differentiation of cells [11, 12]. Ca-ion signaling plays also an important role in the osteoblast differentiation process, being crucial to stimulate osteoblast differentiation and increase osteogenesis by regulating osteocalcin, bone sialoprotein, osteopontin, ALP, and BMP-2 expression in mesenchymal stem cells [13].

Thus, the composition of the adsorbed protein layer plays a pivotal role in the initiation and progress of biological responses occurring after implantation. Proteins forming part of this layer initiate and regulate processes such as potential foreign body response, inflammation, coagulation, and fibrinolysis and even bone cell activity in the earlier stages of osteogenesis [11, 14]. Consequently, the interaction of the biomaterial when exposed to serum proteins can provide preliminary clues to implant designers as to what compositions are more likely to be rejected/accepted by the host, as previously demonstrated in vitro [15, 16] and in vivo [14, 17, 18].

In this work, we aim at evaluating the protein adsorption patterns and in vivo osseointegration at regular Ti implant surfaces compared to surfaces with adsorbed Ca-ions. We hypothesize that differential surface protein adsorption profiles in vitro may lead to differences in bone implant integration in vivo. Among the animal models for implant osseointegration, the rabbit has been widely employed in the past because of its fast skeletal change and human-like mineral density [19, 20]. When the implantation is made in the femoral condyles, the implants and peri-implant tissues are mechanically stimulated within a less dense trabecular bony architecture, which represents a favorable scenario to test more performing implant developments in challenging situations [21].

## Methods

### Substrates

We prepared the surfaces out of machined CP titanium grade IV on two different geometries: (1) 12.7-mm diameter and 1-mm thick discs and (2) custom-made cylindrical implants with a top 2-mm diameter × 4-mm long part and a bottom 4-mm diameter × 2-mm long part (like a T upside-down) for in vivo testing.

The control and Ca-ion surfaces were prepared according to the protocols described in Anitua et al. [4]. Briefly, we roughened the samples’ surfaces by sequential acid etching and further cleaning and conditioning in a class A clean room (BTI Biotechnology Institute S.L., Vitoria, Spain). The control surfaces were no further modified, and the Ca-ion surface was prepared according to a proprietary process (unicCa®) from the control surfaces. Briefly, Ca-ion surfaces were prepared by dip coating during 30 s in a bath containing 5 wt% CaCl_2_ in a clean room class A. We β-ray sterilized all the samples and stored them until use. In addition to these two surfaces, Ca-ion surfaces after immersion for 5 s in deionized water and let air dry (Ca-ion diluted) were prepared to assess the morphology of the topography underlying the hydrated CaCl_2_ layer. We took representative scanning electron microscopy (SEM) micrographs of the surfaces at different magnifications with a Quanta 200FEG SEM (FEI Eindhoven, The Netherlands) at 30 kV acceleration voltage and 3 Å spot size.

### Surface characterization

We used the SEM Phenom Pro-X (Phenom-World BV, Eindhoven, The Netherlands) software (Phenom Pro Suite) to acquire images and quantity of the surface morphology by reconstructing its 3D surface, from which mean surface roughness values (S_a_) were calculated. S_a_ is the arithmetic mean of the absolute deviations of the roughness profile from the mean plane. We applied two cutoff filters: 20×20 nm to 20×20 μm and 10×10 μm to 50×50 μm in order to separate the S_a_ roughness values (S_ar_) and the S_a_ waviness values (S_aw_) respectively from the primary (S_ap_) unfiltered values. Three different areas of 270 μm^2^ of each sample were selected for 3D reconstruction and calculation. Results were averaged from three measurements per surface condition and substrate geometry.

To analyze the composition of Ca-ion and control surfaces, we used the energy dispersive X-ray spectroscopy (EDS) equipped in the SEM Phenom Pro-X. The unit has a thermoelectrically cooled silicon drift detector and a narrow Si_3_N_4_ window for elemental detection. We scanned areas of 270 μm^2^ at ×1000 magnification and 15 kV acceleration voltage to maximize EDS yield. We used three samples per surface type and geometry.

### Adsorbed protein layer

We incubated the control and Ca-ion samples in a 24-well NUNC plates (Thermo Fisher Scientific, Waltham, MA, USA) for 3 h (37 °C, 5% CO_2_) with 1 mL of human blood serum from male AB plasma (Sigma–Aldrich, Merck KGaA, Darmstadt, Germany). In order to allow a standard, replicable characterization, we used commercial human serum as previously described in Romero-Gavilán et al. [22]. After 3 h incubation, we removed the serum and washed the discs five times with ddH_2_O and once with 100 mM NaCl, 50 mM Tris–HCl (pH 7.0) to eliminate non-adsorbed proteins. We collected the adsorbed protein layer by washing the discs with an elution (0.5 M triethylammonium bicarbonate buffer (TEAB), 4% of sodium dodecyl sulfate, 100 mM of dithiothreitol (DTT)). We carried out four independent experiments for each type of surface, and we used four discs of each surface type in each experiment. We used a Pierce BCA assay kit (Thermo Fisher Scientific, Waltham, MA, USA) to quantify the serum protein content, which was 50 μg/μL.

### Proteomic analysis

We performed the proteomic analysis as described by Romero-Gavilán et al. [22] with minor variations. Briefly, we digested the eluted protein in-solution, following the FASP protocol established by Wiśniewski et al. [23] and loaded onto a nanoACQUITY UPLC system connected online to an LTQ Orbitrap XL ETD (Thermo).

We analyzed each surface in quadruplicate. We used the Progenesis software (Nonlinear Dynamics, Newcastle, UK) to perform the differential protein analysis using as described before [24]. We used the DAVID GO (https://david.ncifcrf.gov/) and Panther classification system (http://www.pantherdb.org/) for the functional annotation of the proteins.

### Surgical procedure

We used nine implants (*n*=9) per surface type (control and Ca-ion) and defined the evaluation time at 2 and 8 weeks post implantation. We inserted the implants bilaterally in the medial femoral condyle of 18 New Zealand white female rabbits. The rabbits were skeletally mature, aged 23 ± 2 weeks, and weighed 3.2±0.7 kg. Following sedation and anesthesia, we administered a preoperative antibiotic. We made the incision through the skin, the muscular fascia, and sartorius muscle, exposing the superior distal quadrant of the medial condyle for implantation. To prepare the implant sites, we used drills of 2.5 and 4.2 mm under thorough saline irrigation. Prior to implant installation by press-fit, we cleaned the implant site from drilling remnants. We sutured the tissues in layers. After surgery, the rabbits received analgesia (Metacam, 0.2 mg/kg, subcutaneous) and antibiotics (cefazoline 0.2 mg/kg, intramuscular) for 4 days. We monitored on a daily basis the animals’ weight, behavior, and health conditions. After 2 and 8 weeks of implantation, the animals were euthanized.

We performed all procedures following the ISO 10993-6:2016 (Annex D). We handled the animals and performed the surgeries according to the directive of the European Parliament and Council of the European Communities (2010/63/EU) and the Spanish legislation (RD 1201/2005 and Law 32/2007). The ethics committee of the Autonomous Government of Aragón (Spain) approved the protocol of this study and certified the fulfillment of animal welfare guidelines (file number PI26/12). The study has been carried out in compliance with the ARRIVE EQUATOR guidelines.

### Histological evaluation and histomorphometry analysis

After sacrifice, we extracted the condyles and fixed the implants with the surrounding bone in 4% buffered formalin solution for at least 24 h. The condyles with the implants were dehydrated in ethanol from growing concentrations from 70 to 100% and embedded in a light-curing acrylic resin (Technovit 7200 VLC, Heraeus-Kulzer, Wehrheim, Germany) according to the manufacturer’s instructions. Following polymerization, we cut the blocks to a thickness of 300 μm and polished them to their final thickness. We got two non-decalcified 20-μm-thick sections of the implants following their longitudinal axis using a diamond microtome saw (Exakt Technologies, Oklahoma City, USA). We stained the sections with Harris hematoxylin and Wheatley’s trichromatic stain and examined them at different magnifications with a Leica DMLB light microscope (Leica Microsystems, Wetzlar, Germany) coupled to a Leica DFC300FX digital camera. The ground sections were observed at ×2.5, ×5, and ×20.

We performed a blind histomorphometry analysis to quantify the bone response and osseointegration around the 4-mm diameter × 2-mm long bottom part of the implants. The top part of the implant was discarded in order to prevent undesired data noise coming from slightly different implant placement heights and from the more variable regenerative situation near the soft tissues. We took the measurements with a ×5 objective, and we calculated the bone to implant contact (BIC) and bone volume density (BVD) percentages with the software ImageJ 1.47 (National Institutes of Health, Bethesda, MD, USA). BIC refers to the contour of direct bone-implant contact without interposition of fibrous tissue and BVD to the area occupied by bone tissue in the 1 mm region closer to the surface.

### Statistical analysis

We confirmed data normality prior to comparisons (Shapiro–Wilk) and expressed them as mean ± standard deviation (SD). We determined the differences between the means by two-sample independent Student’s two-tailed homoscedastic *t*-test between surfaces. We considered statistical significance for *p*<0.05. We used Origin v7.5 (OriginLab Corporation, Northampton, MA, USA) for all statistical analyses except for the proteomic data, for which we used the Progenesis QI software. We considered differential protein adsorption for *p*<0.05 and a ratio higher than 1.5 in either direction.

## Results

### The implant surfaces

**Fig. 1:**
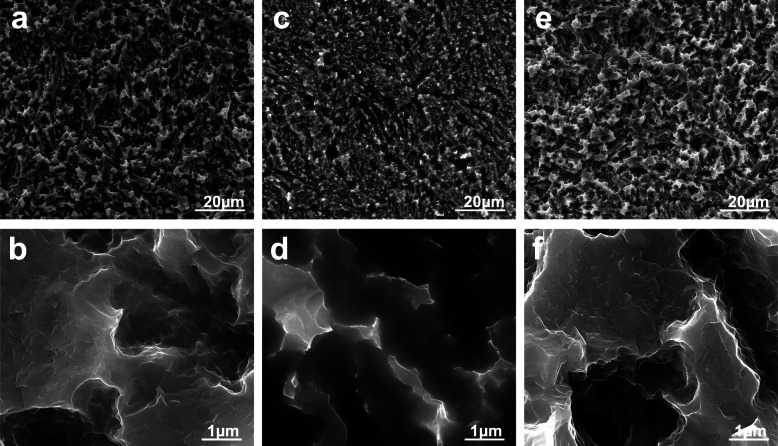
Representative SEM micrographs of the control (**a**, **b**) and the and Ca-ion surfaces before (**c**, **d**) and after dilution (**e**, **f**). Magnifications ×2500 (**a**, **c**, **e**) and ×40,000 (**b**, **d**, **f**)

Figure 1 shows representative SEM images of the control (a, b) and Ca-ion before (c, d) and after dilution (e, f). The Ca-ion-diluted surface is similar to the control: both show the typical micron (a, e) and sub-micron (b, f) surface features of these implant surface preparations. At the Ca-ion surface (c, d), the vacuum produced in the chamber of the SEM dehydrates the CaCl_2_ layer and resembles a coating embedded within the surface roughness.

Table 1 shows the unfiltered (S_ap_), roughness (S_ar_), and waviness (S_aw_) filtered topographical parameters of the surfaces. The control and the Ca-ion-diluted surfaces show no significant differences in any roughness values, while the dehydrated deposit of CaCl_2_ inside the SEM chamber fills the pits of the roughness and produces thus a significant reduction in all roughness values with respect to the control or the Ca-ion diluted.

**Table 1:** Roughness of control and Ca-ion surfaces before and after dilution. Data is shown in nm ± standard deviation

	Control	Ca-ion	Ca-ion diluted
S_ap_	2320 ± 242	1080 ± 92	2040 ± 259
S_ar_	1021 ± 122	612 ± 70	1069 ± 144
S_aw_	1553 ± 172	678 ± 56	1423 ± 215

**Fig. 2:**
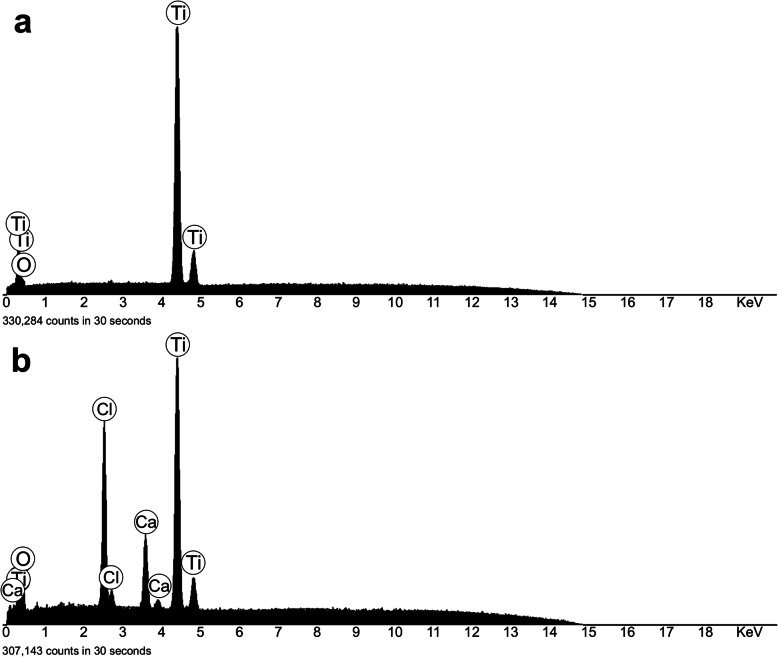
EDS spectra of the control (**a**) and the Ca-ion (**b**) surfaces

EDS spectra corresponding to the control surfaces (Fig. 2) yielded 68.5±5.3 At% associated with titanium and 31.6±7.3 At% associated with oxygen. Carbon was below the detection limit, typically below 2 At%. Ca-ion surfaces’ spectra contained 48.4± 5.2 At% oxygen, 32.4±4.8 At% titanium, 12.8±2.2 At% chlorine, and 6.6±2.3 At% calcium.

### The protein adsorption

We analyzed the eluted proteins by LC-MS/MS and performed a comparative analysis between the data for each surface with Progenesis. Table 2 shows the main results of the analysis carried out comparing control and Ca-ion surfaces. Raw data is shown in Table S1. Seventeen proteins were differentially absorbed by the Ca-ion surface, wherein four were more absorbed and 13 presented less affinity with this material. The surface treatment led to a significant increase of one protein related with coagulation (FA10) and three related with immune responses (LYSC, PIP, and SAMP). Five proteins related with coagulation processes (A1AT, PLMN, FA12, KNG1, and HEP2) and three related with immune responses (VTNC, SAA4, and CFAH) were less adsorbed at Ca-ion surfaces. APOE, which plays an important role in bone metabolism by allowing the entry of vitamin K into the osteoblasts to carry out the carboxylation of osteocalcin, was found in highest proportion attached onto Ti. Also, TRFE, a protein, linked to the anchoring and transport of Fe^3+^, DHX8, with functions in RNA processing and two proteins related to ATP synthesis (ATPA and ACTBL).

**Table 2:** Differential Ca-ion/control adsorbed protein ratio. Data with ANOVA *p* < 0.05 and a ratio higher than 1.5 in either direction was considered as significantly different

Accession	Description	***p*** value	Ratio
FA10_HUMAN	Coagulation factor X	1.13E−04	181.42
LYSC_HUMAN	Lysozyme C	2.29E−02	13.60
PIP_HUMAN	Prolactin-inducible protein	3.90E−02	2.98
SAMP_HUMAN	Serum amyloid P-component	4.66E−02	2.59
A1AT_HUMAN	Alpha-1-antitrypsin	3.97E−03	0.53
TRFE_HUMAN	Serotransferrin	1.69E−02	0.46
VTNC_HUMAN	Vitronectin	2.13E−02	0.45
APOE_HUMAN	Apolipoprotein E	4.78E−03	0.41
SAA4_HUMAN	Serum amyloid A-4 protein	3.45E−02	0.38
PLMN_HUMAN	Plasminogen	1.10E−02	0.28
FA12_HUMAN	Coagulation factor XII	4.26E−02	0.26
KNG1_HUMAN	Kininogen-1	1.16E−04	0.24
ATPA_HUMAN	ATP synthase subunit alpha mitochondrial	3.56E−02	0.24
ACTBL_HUMAN	Beta-actin-like protein 2	3.65E−02	0.19
DHX8_HUMAN	ATP-dependent RNA helicase DHX8	4.11E−02	0.13
HEP2_HUMAN	Heparin cofactor 2	4.92E−02	0.13
CFAH_HUMAN	Complement factor H	1.28E−02	0.06

The DAVID and Panther systems were used to associate the adsorbed proteins with their functions in distinct biological pathways (Figure S1). In control surfaces (a), we identified proteins associated with ATP synthesis, plasminogen-activating cascade, Huntington and Alzheimer diseases, inflammation mediated by chemokine and cytokine, integrin and cadherin signaling pathways, cytoskeletal regulation, and blood coagulation. In Ca-ion surfaces (b), the only function identified was blood coagulation.

### In vivo osseointegration

Because of exitus of rabbit number 13, two implants, one of each surface, were not available for analysis. The post-operative period was uneventful for all remaining rabbits. We analyzed then nine implants per surface type at 2 weeks and eight implants at 8 weeks. Supplementary tables 2, 3, 4 and 5 show all the data obtained.

**Fig. 3:**
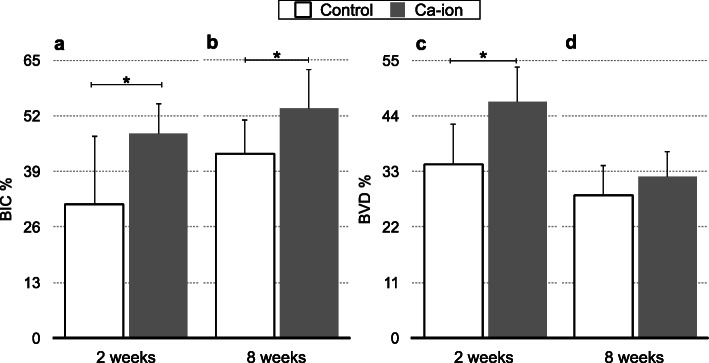
Bone implant contact (BIC; **a** and **b**) and bone volume density (BVD; **c** and **d**) in percentage (%) of control and Ca-ion surfaces after 2 and 8 weeks of implantation from two ground sections of each of the 36 implants placed in 18 rabbits. Results are shown as mean ± SD

Figure 3 shows the results of the bone implant contact (BIC) and bone volume density (BVD) of the control and Ca-ion surfaces at 2 and 8 weeks after implants placement. At 2 weeks of healing, the BIC percentage of the control and the Ca-ion surfaces was 31.4±16.5% and 47.9±7.6%, respectively (*p*=0.016, Table S2); the BVD% was 34.4±8.2% and 46.6±7.0%, respectively (*p*=0.004, Table S4). At 8 weeks, the BIC percentage increased to 43.2±8.1% and 53.8±9.5%, respectively (*p*=0.028, Table S3), but the BVD percentage decreased to 28.3±6.5% and 32.0±5.0%, respectively (*p*=0.195, Table S5).

**Fig. 4:**
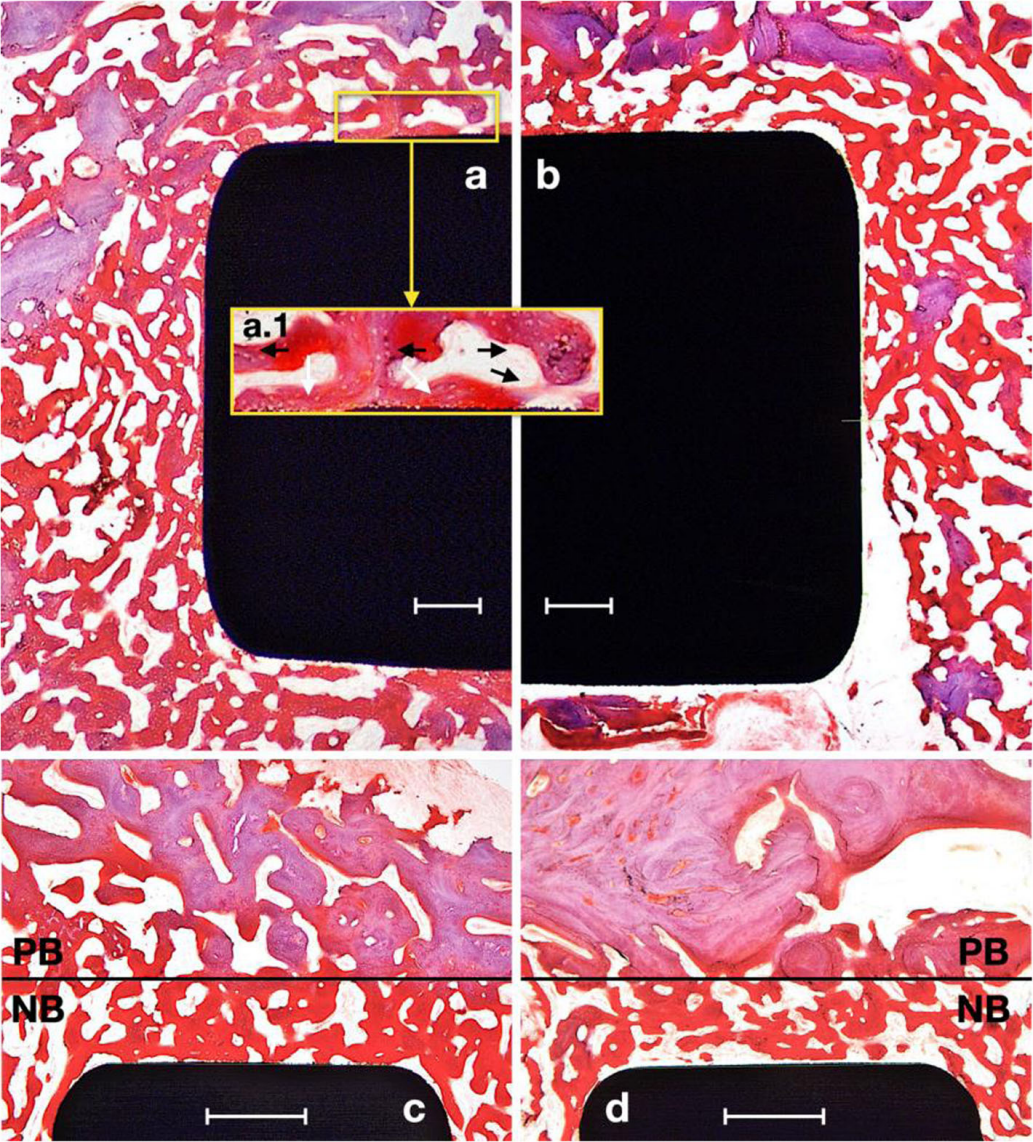
Harris hematoxylin and Wheatley’s trichrome-stained ground sections of Ca-ion (**a**, inset a.1, **c**) and control (**b**, **d**) implant surfaces at 2 weeks of healing. PB, parent bone, NB, new bone. Scale bars 500 μm

**Fig. 5:**
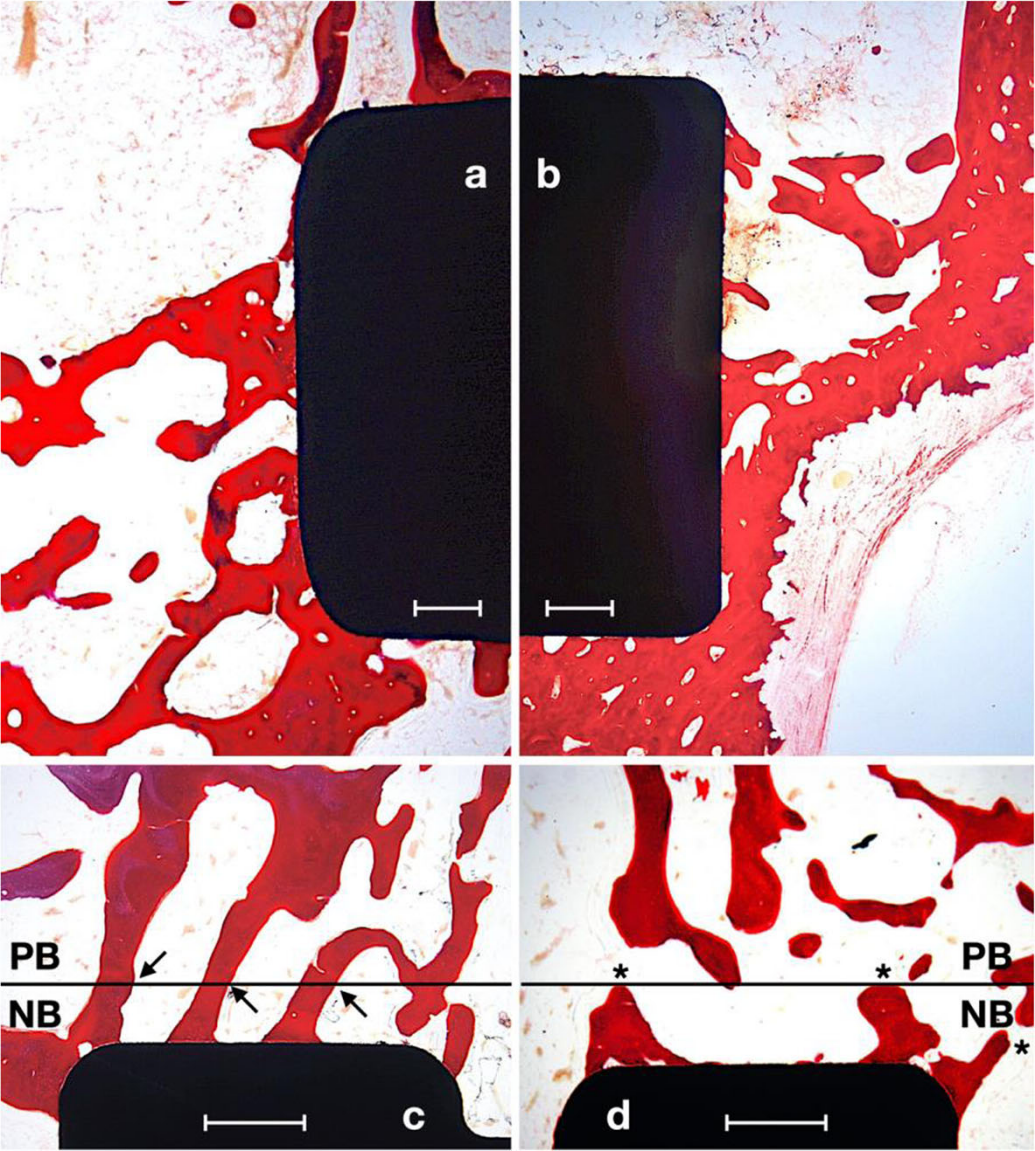
Harris hematoxylin and Wheatley’s trichrome-stained ground sections of Ca-ion (**a**, **c**) and control (**b**, **d**) implant surfaces at 8 weeks of healing. PB, parent bone; NB, new bone. Scale bars 500 μm

Figures 4 and 5 show representative histological ground sections of the implants. The histological evaluation showed no sign of inflammatory response or complication, and all implants osseointegrated correctly. The Harris hematoxylin and Wheatley’s trichromatic stain showed immature unorganized bone with elevated osteoblastic activity in red tones, while light purple staining showed the parent mature bone.

At 2 weeks, we observed high bony activity near the surfaces (Fig. 4). There was a clear separation (marked with a black line) between the drilled region near the implant surface, in which new bone (NB) formation was taking place, and the parent bone (PB) with the pre-existing bony architecture (Fig. 4 c and d). We found poorly mineralized bone in the NB area (bright red staining) with high osteoblastic activity in the marginal areas and near the implant surfaces (Fig. 4a.1). White arrows in a.1 show high osteoblastic activity at the implant surface, and black arrows show bluish staining corresponding to newly mineralized bone. The area above the line shows the parent old bone (PB). Parent bone showed osteonal structures, lamellar arrangement, and higher mineralization.

At 8 weeks of healing (Fig. 5), the bone contacting the surfaces remained but decreased the density in the 1 mm region. Remodeling led to the formation of trabeculae linked to the parent subchondral bone, mostly taking place in Ca-ion surfaces. An example of this is marked with black arrows in Fig. 5c. Conversely, an example of newly formed bone near the surfaces not linked to the parent bone trabeculae is marked with asterisks in Fig. 5d.

## Discussion

Upon implant placement, adsorption of proteins onto the surface takes place immediately [25, 26]. The characteristics of the protein corona formed at the surface depend on the characteristics of the surface itself, conditioning the evolution of the newly formed interface and the implantation outcome. Ca-ions are known to interact electrostatically with the oxide layer of Ti and promote the advent of negatively charged protein residues to the surface [27]. Surface hydrophilicity is known to increase the adsorption of fibrinogen and fibrin [28]. Nanotopography, in addition, stimulates platelet activation [29, 30].

The implant surfaces studied present both micron and submicron topographical features. It has been previously reported that additional nanofeatures emerge at Ca-ion surfaces [26]. SEM visualization requires high vacuum conditions, which modifies the physical state of the Ca-ion surfaces with respect to ambient conditions, in which Ca and Cl ions are dissociated and mostly released from the surface within the first seconds of exposure to polar liquids such as water or blood [26]. SEM images of the Ca-ion washed surfaces showed that the underlying topography remains unaltered. But when exposed to blood plasma, Ca-ion surfaces have been reported to induce surface clot formation, platelet adsorption, and activation [4]. Activated platelets release both osteogenic and angiogenic factors, calcium, and phospholipids of the platelet membrane [8, 25, 31]. The blood clot at the implant surface helps retain these factors, vectoring osteoprogenitor cell recruitment until complete fibrinolysis takes place [32]. Osteoprogenitor cells create a stable extracellular matrix for new bone formation in contact with the implant surface [26].

Titanium superior osseointegration has been related to its strong thrombogenicity [33, 34]. Ca ions are cofactor intermediators in a number of events of the coagulation cascade [9]. Considering this, here we sought to improve our understanding on how calcium ions at titanium implant surfaces contribute to accelerate the rate of implant osseointegration from a surface proteomic viewpoint.

The experiments conducted by Hong et al. [35] associated the higher thrombogenicity of titanium oxides to the generation of high levels of activated FA12. The serine protease FA12 starts the contact system or intrinsic pathway of coagulation, preferentially in contact with negatively charged surfaces such as titanium oxides [36]. Hong and coworkers also suggested that titanium-regulated FA12 in a particular way with respect to other thrombogenic metal oxides. Our proteomic experiments showed that the standard titanium surfaces used as control adsorb nearly four times more FA12 than Ca-ion surfaces. Concomitantly FA12 activation triggers the kallikrein system in an amplification loop. By-products of this process, such as bradykinin, have been reported to induce inflammation, thrombotic reactions, cell proliferation, and vascular permeability [37, 38]. Conversely, Ca-ion surfaces adsorbed over 180 times more FA10 than the control surfaces. FA10, also known as Stuart–Prower factor, is the first factor of the common path of coagulation. FA10 acts by cleaving prothrombin and, with Ca ions, activates thrombin and allows the formation of the fibrin clot [25]. Coagulation at Ca-ion surfaces is thus governed by the common pathway of coagulation, probably minimizing the contact activation and amplification system that takes place at regular Ti surfaces. In other words, Ca-ion surfaces are more thrombogenic than regular titanium surfaces and the mechanisms of coagulation involved belong to the later stages of the cascade, suggesting lower activation of the complement cascade due to competition with the coagulation factors. Remarkably, abundant serum proteins, such as albumin or fibronectin, were not differentially adsorbed on the surfaces. An explanation for this may be given according to the Vroman effect, which showed that even though a protein is abundant in the medium, it may have less affinity for a surface than less abundant ones [5].

We used the same surface samples for protein adsorption and bone-integration studies. The only difference being the presence or absence of surface Ca-ions. In this work, we obtained similar osseointegration indexes to those previously published in the rabbit [4]. Compared to that study, we reported better BIC results of Ca ion surfaces already at 2 weeks of healing. There, the implant shape was threaded and more invasive, while here we used a smaller, non-threaded pin with no intended gap between the implant surfaces and the implantation site. This simpler geometry, closer to the parent bone, may have been more favorable for the development of the regenerative scaffold of fibrin, platelets, and growth factors at the surface bridging the gap between the results obtained with or without blood plasma application [4].

The differences found in osseointegration between Ca-ion surfaces and regular surfaces can be associated with the differential mechanisms of formation of the clot around the two implant surfaces studied. The fibrin structure of the peri-implant clot affects osteoconduction and new bone formation on implant surfaces [12]. The quick formation of a surface-bound stable clot at Ca-ion surfaces acts as a chemotactic scaffold for the continuous recruitment and migration of osteogenic cells, which is a prerequisite for new bone formation starting at the implant surface [13, 26, 39, 40]. The structural proteins of the clot, with embedded growth factors, serve as physical support for cell adhesion and function, creating a more osteoconductive microenvironment [31]. Indeed, Ca-ion prothrombotic surfaces created more bone at only 2 weeks of healing but also established earlier connections to the parent bone. The lateral stabilization of the implant is fundamental for the functional prosthetic loading. In a study with representative bacterial strains of the oral cavity leading to pervasive infections and in the presence of human serum and salivary proteins, Ca-ion surfaces showed a significant reduction in adhesion and biofilm formation [41]. These results and the more osteogenic microenvironment generated by Ca-ion modification at the implant surface may lead to greater implant stability in the long term.

The decrease in bone volume density in 1 mm around both implants at 8 weeks of implantation merits a discussion too. We found similar results using this animal model in the past [4]. Woven unorganized bone around the implants converts progressively into trabeculae that join the implant surface with the subchondral bone. Remodeling gives rise to defined trabeculae. Therefore, in both cases, the volume of bone around the implants decreases to the areas covered by the forming trabeculae that stabilize the implants laterally. In other terms, there is seemingly less bone, but it is mechanically more valuable. Indeed, the bone implant contact increases because there is more bone covering the surfaces, extending the trabeculae implant-end thickness. Concomitantly there is less or no unorganized bone or bone debris in the 1 mm region around the implants, which has no relevant mechanical role.

We found no acute inflammatory signals at either control or Ca-ion surfaces, even though both surfaces displayed a distinct set of proteins related to the innate immune system: VTNC, SAA4, and CFAH at control Ti; LYSC, PIP, and SAMP at Ca-ion. SAMP comprises five subunits with two Ca-ion-binding positions and has been related to immunological responses [42]. The proteomics analysis was performed after incubation of the surfaces for 3 h, for this period of incubation is enough for the stabilization of the protein corona at regular titanium surfaces. However, the Ca-ion-modified surface releases calcium ions over longer periods of time [4, 26], varying thereby the chemical characteristics at the interface and thus possibly the proteins adsorbed. To overcome this limitation, longer protein adsorption studies are underway. Changes in the calcium ion content of the surface over time are expected to alter in turn the protein composition of the interface and will help to make better understanding of its relationship with the differential osteogenic functions observed in vivo. The differences detected in protein adsorption may be an explanation for the different regeneration mechanisms found in the surfaces studied. From a strict scientific viewpoint, there seems to be a correlation between human protein adsorption and rabbit osseointegration. Therefore, the authors plan to pursue the experimentation unifying species and performing more in-depth analysis to clarify a potential causal relationship.

## Conclusion

Ca-ion surfaces adsorbed overwhelmingly FA10, signaling an advanced stage of the coagulation cascade and suggesting a strong prothrombotic reaction at these surfaces. In vivo, Ca-ion surfaces stimulated more bone formation around the implants than the standard implant surfaces used as controls. These results are especially relevant for the low quantity or quality of bone situations. Short or narrow implants with limited contacting surfaces can be particularly benefited, paving the way to less invasive surgeries, shorter healing times, and overall lower intervention costs. Taken together, these results shed more light in the relationship between surface clot formation and implant osseointegration.

## Supplementary Information

**Additional file 1: Table S1.** Differential Ca-ion/Control adsorbed proteins. Data with ANOVA *p* < 0.05 and a ratio higher than 1.5 in either direction was considered as significantly different. The detected amount of protein is showed as log2-transformed normalized abundance values. The data obtained through the analysis of the four independent replicates for each sample were described as n_1-4_.

**Additional file 2: Figure S1.** Panther diagram of the pathways associated with the proteins adhered differentially to Control (a) and Ca-ion (b) surfaces, respectively.

**Additional file 3: Table S2.** Bone implant contact (BIC) in percentage (%) of Control and Ca-ion surfaces after 2 weeks of implantation from two ground sections (GS) of each of the 18 implants placed in 9 rabbits. Results are shown as mean ± SD.

**Additional file 4: Table S3.** Bone implant contact (BIC) in percentage (%) of Control and Ca-ion surfaces after 8 weeks of implantation from two ground sections (GS) of each of the 16 implants placed in 8 rabbits. Results are shown as mean ± SD.

**Additional file 5: Table S4.** Bone volume density (BVD) in percentage (%) of Control and Ca-ion surfaces after 2 weeks of implantation from two ground sections (GS) of each of the 18 implants placed in 9 rabbits. Results are shown as mean ± SD.

**Additional file 6: Table S5.** Bone volume density (BVD) in percentage (%) of Control and Ca-ion surfaces after 8 weeks of implantation from two ground sections (GS) of each of the 16 implants placed in 8 rabbits. Results are shown as mean ± SD.
