# Supplementary material for: Influence of calcium ion-modified implant surfaces in protein adsorption and implant integration
Source: Int J Implant Dent. 2021 Apr 21;7:32. doi: 10.1186/s40729-021-00314-1 (PMC8058122; doi:10.1186/s40729-021-00314-1)
Supplement: Supplementary file 1 — Additional file 1: Table S1. Differential Ca-ion/Control adsorbed proteins. Data with ANOVA p < 0.05 and a ratio higher than 1.5 in either direction was considered as significantly different. The detected amount of protein is showed as log2-transformed normalized abundance values. The data obtained through the analysis of the four independent replicates for each sample were described as n1-4. [file 40729_2021_314_MOESM1_ESM.docx]

| **Normalized abundance** | | | | | | | |  |  |  |  |
| --- | --- | --- | --- | --- | --- | --- | --- | --- | --- | --- | --- |
| **Ca-ion** | | | | **Control** | | | | **Ca-ion/Control** | |  |  |
| **Accession** | **Description** | **n_1_** | **n_2_** | **n_3_** | **n_4_** | **n_1_** | **n_2_** | **n_3_** | **n_4_** | **p value** | **Ratio** |
| **FA10_HUMAN** | Coagulation factor X | 21.28 | 21.19 | 21.48 | 18.45 | 13.35 | 13.39 | 13.83 | 11.82 | **1.13E-04** | **181.42** |
| **LYSC_HUMAN** | Lysozyme C | 17.36 | 17.08 | 19.28 | 19.50 | 14.94 | 16.44 | 15.30 | 11.49 | **2.29E-02** | **13.60** |
| **PIP_HUMAN** | Prolactin-inducible protein | 18.04 | 17.14 | 17.30 | 17.15 | 15.44 | 16.32 | 17.07 | 14.48 | **3.90E-02** | **2.98** |
| **SAMP_HUMAN** | Serum amyloid P-component | 24.14 | 24.13 | 24.70 | 22.30 | 22.22 | 22.81 | 22.10 | 22.66 | **4.66E-02** | **2.59** |
| **A1AT_HUMAN** | Alpha-1-antitrypsin | 17.42 | 17.37 | 18.02 | 17.19 | 18.30 | 18.25 | 18.64 | 18.47 | **3.97E-03** | **0.53** |
| **TRFE_HUMAN** | Serotransferrin | 20.92 | 21.77 | 21.92 | 21.07 | 22.18 | 23.16 | 22.16 | 22.71 | **1.69E-02** | **0.46** |
| **VTNC_HUMAN** | Vitronectin | 24.46 | 23.99 | 24.31 | 23.07 | 24.50 | 25.33 | 25.24 | 25.38 | **2.13E-02** | **0.45** |
| **APOE_HUMAN** | Apolipoprotein E | 25.05 | 24.53 | 25.49 | 24.50 | 25.68 | 26.41 | 26.43 | 26.18 | **4.78E-03** | **0.41** |
| **SAA4_HUMAN** | Serum amyloid A-4 protein | 19.07 | 18.51 | 20.17 | 18.34 | 19.64 | 21.07 | 20.59 | 20.34 | **3.45E-02** | **0.38** |
| **PLMN_HUMAN** | Plasminogen | 17.67 | 18.69 | 18.95 | 17.24 | 19.22 | 20.42 | 19.77 | 20.46 | **1.10E-02** | **0.28** |
| **FA12_HUMAN** | Coagulation factor XII | 19.40 | 19.17 | 19.74 | 16.65 | 19.96 | 20.88 | 21.38 | 20.60 | **4.26E-02** | **0.26** |
| **KNG1_HUMAN** | Kininogen-1 | 19.37 | 19.01 | 19.18 | 18.39 | 21.22 | 20.95 | 21.09 | 20.82 | **1.16E-04** | **0.24** |
| **ATPA_HUMAN** | ATP synthase subunit alpha mitochondrial | 12.34 | 12.86 | 14.82 | 11.41 | 15.58 | 14.38 | 15.19 | 14.59 | **3.56E-02** | **0.24** |
| **ACTBL_HUMAN** | Beta-actin-like protein 2 | 12.12 | 15.75 | 12.55 | 13.70 | 15.00 | 16.09 | 16.68 | 15.84 | **3.65E-02** | **0.19** |
| **DHX8_HUMAN** | ATP-dependent RNA helicase DHX8 | 17.79 | 17.95 | 15.82 | 13.23 | 19.22 | 18.66 | 19.55 | 18.97 | **4.11E-02** | **0.13** |
| **HEP2_HUMAN** | Heparin cofactor 2 | 11.97 | 16.18 | 16.17 | 12.47 | 18.18 | 16.84 | 17.50 | 16.26 | **4.92E-02** | **0.13** |
| **CFAH_HUMAN** | Complement factor H | 10.81 | 10.79 | 14.78 | 12.67 | 17.12 | 17.70 | 14.62 | 15.97 | **1.28E-02** | **0.06** |

Table S 1 Differential Ca-ion/Control adsorbed proteins. Data with ANOVA p < 0.05 and a ratio higher than 1.5 in either direction was considered as significantly different. The detected amount of protein is showed as log2-transformed normalized abundance values. The data obtained through the analysis of the four independent replicates for each sample were described as n_1-4_.
