# Supplementary figures and images for: Influence of calcium ion-modified implant surfaces in protein adsorption and implant integration
Source: Int J Implant Dent. 2021 Apr 21;7:32. doi: 10.1186/s40729-021-00314-1 (PMC8058122; doi:10.1186/s40729-021-00314-1)

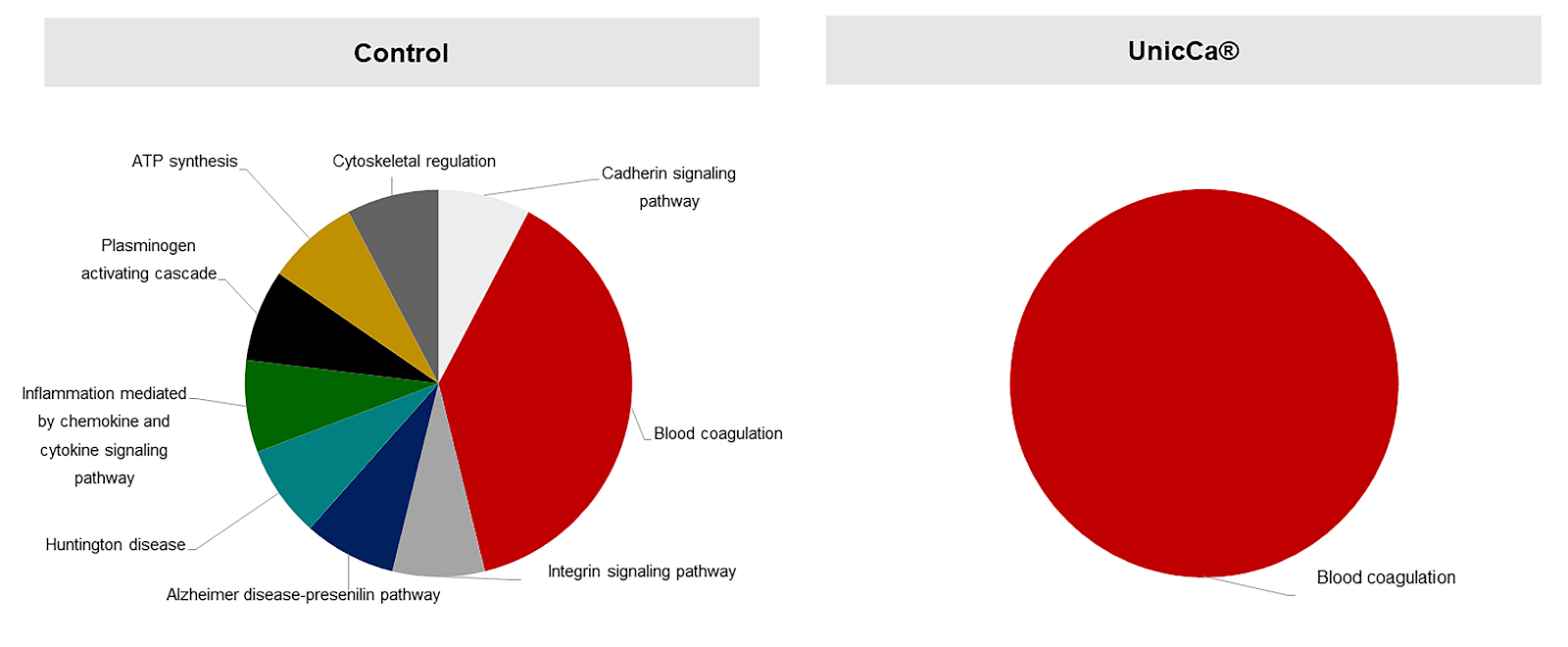

Supplement: Supplementary file 2 — Additional file 2: Figure S1. Panther diagram of the pathways associated with the proteins adhered differentially to Control (a) and Ca-ion (b) surfaces, respectively. [file 40729_2021_314_MOESM2_ESM.docx]
