# Supplementary material for: Influence of calcium ion-modified implant surfaces in protein adsorption and implant integration
Source: Int J Implant Dent. 2021 Apr 21;7:32. doi: 10.1186/s40729-021-00314-1 (PMC8058122; doi:10.1186/s40729-021-00314-1)
Supplement: Supplementary file 3 — Additional file 3: Table S2. Bone implant contact (BIC) in percentage (%) of Control and Ca-ion surfaces after 2 weeks of implantation from two ground sections (GS) of each of the 18 implants placed in 9 rabbits. Results are shown as mean ± SD. [file 40729_2021_314_MOESM3_ESM.docx]

| 2 weeks | Control | | | Ca-ion | | |
| --- | --- | --- | --- | --- | --- | --- |
| BIC | GS 1 | GS 2 | Mean | GS 1 | GS 2 | Mean |
| 1 | 28.42% | 30.53% | 29.48% | 45.82% | 43.79% | 44.80% |
| 2 | 2.78% | 7.13% | 4.96% | 39.43% | 45.44% | 42.43% |
| 3 | 50.10% | 49.18% | 49.64% | 48.34% | 53.90% | 51.12% |
| 4 | 5.75% | 8.59% | 7.17% | 48.77% | 50.62% | 49.69% |
| 5 | 39.05% | 44.54% | 41.80% | 38.10% | 42.87% | 40.49% |
| 6 | 32.36% | 41.69% | 37.03% | 46.13% | 49.87% | 48.00% |
| 7 | 38.60% | 38.08% | 38.34% | 61.83% | 51.17% | 56.50% |
| 8 | 49.04% | 53.54% | 51.29% | 35.65% | 40.37% | 38.01% |
| 9 | 22.86% | 22.47% | 22.66% | 57.79% | 61.73% | 59.76% |
| Mean | 29.88% | 32.86% | 31.37% | 46.87% | 48.86% | 47.87% |
| SD | 16.99% | 16.96% | 16.54% | 8.72% | 6.57% | 7.56% |

Table S 2 Bone implant contact (BIC) in percentage (%) of Control and Ca-ion surfaces after 2 weeks of implantation from two ground sections (GS) of each of the 18 implants placed in 9 rabbits. Results are shown as mean ± SD.
