# Supplementary material for: Influence of calcium ion-modified implant surfaces in protein adsorption and implant integration
Source: Int J Implant Dent. 2021 Apr 21;7:32. doi: 10.1186/s40729-021-00314-1 (PMC8058122; doi:10.1186/s40729-021-00314-1)
Supplement: Supplementary file 4 — Additional file 4: Table S3. Bone implant contact (BIC) in percentage (%) of Control and Ca-ion surfaces after 8 weeks of implantation from two ground sections (GS) of each of the 16 implants placed in 8 rabbits. Results are shown as mean ± SD. [file 40729_2021_314_MOESM4_ESM.docx]

| 8 weeks | Control | | | Ca-ion | | |
| --- | --- | --- | --- | --- | --- | --- |
| BIC | GS 1 | GS 2 | Mean | GS 1 | GS 2 | Mean |
| 10 | 40.36% | 41.91% | 41.13% | 47.59% | 45.54% | 46.57% |
| 11 | 43.98% | 41.46% | 42.72% | 49.05% | 54.37% | 51.71% |
| 12 | 58.89% | 54.47% | 56.68% | 54.39% | 64.09% | 59.24% |
| 14 | 23.25% | 33.51% | 28.38% | 50.49% | 49.91% | 50.20% |
| 15 | 41.68% | 37.44% | 39.56% | 34.44% | 38.54% | 36.49% |
| 16 | 42.49% | 49.28% | 45.88% | 59.61% | 61.15% | 60.38% |
| 17 | 46.54% | 46.64% | 46.59% | 63.41% | 64.99% | 64.20% |
| 18 | 44.58% | 44.27% | 44.43% | 66.20% | 57.21% | 61.70% |
| ~~13~~ |  |  |  |  |  |  |
| Mean | 42.72% | 43.62% | 43.17% | 53.15% | 54.47% | 53.81% |
| SD | 9.76% | 6.63% | 8.07% | 10.17% | 9.33% | 9.45% |

Table S 3 Bone implant contact (BIC) in percentage (%) of Control and Ca-ion surfaces after 8 weeks of implantation from two ground sections (GS) of each of the 16 implants placed in 8 rabbits. Results are shown as mean ± SD.
