# Supplementary material for: Influence of calcium ion-modified implant surfaces in protein adsorption and implant integration
Source: Int J Implant Dent. 2021 Apr 21;7:32. doi: 10.1186/s40729-021-00314-1 (PMC8058122; doi:10.1186/s40729-021-00314-1)
Supplement: Supplementary file 5 — Additional file 5: Table S4. Bone volume density (BVD) in percentage (%) of Control and Ca-ion surfaces after 2 weeks of implantation from two ground sections (GS) of each of the 18 implants placed in 9 rabbits. Results are shown as mean ± SD. [file 40729_2021_314_MOESM5_ESM.docx]

| 2 weeks | Control | | | Ca-ion | | |
| --- | --- | --- | --- | --- | --- | --- |
| BVD | GS 1 | GS 2 | Mean | GS 1 | GS 2 | Mean |
| 1 | 46.47% | 43.34% | 44.91% | 45.57% | 39.84% | 42.70% |
| 2 | 25.23% | 24.17% | 24.70% | 42.10% | 43.30% | 42.70% |
| 3 | 31.65% | 31.62% | 31.64% | 52.68% | 54.95% | 53.82% |
| 4 | 20.99% | 23.47% | 22.23% | 46.14% | 41.97% | 44.05% |
| 5 | 44.99% | 47.93% | 46.46% | 45.39% | 48.80% | 47.10% |
| 6 | 37.42% | 38.82% | 38.12% | 54.29% | 55.60% | 54.94% |
| 7 | 36.94% | 31.95% | 34.44% | 51.91% | 50.82% | 51.37% |
| 8 | 36.95% | 37.63% | 37.29% | 49.70% | 50.91% | 50.31% |
| 9 | 28.56% | 30.87% | 29.72% | 30.24% | 34.58% | 32.41% |
| Mean | 34.36% | 34.42% | 34.39% | 46.45% | 46.75% | 46.60% |
| SD | 8.55% | 8.24% | 8.15% | 7.28% | 7.20% | 7.02% |

Table S 4 Bone volume density (BVD) in percentage (%) of Control and Ca-ion surfaces after 2 weeks of implantation from two ground sections (GS) of each of the 18 implants placed in 9 rabbits. Results are shown as mean ± SD.
