# Supplementary material for: Influence of calcium ion-modified implant surfaces in protein adsorption and implant integration
Source: Int J Implant Dent. 2021 Apr 21;7:32. doi: 10.1186/s40729-021-00314-1 (PMC8058122; doi:10.1186/s40729-021-00314-1)
Supplement: Supplementary file 6 — Additional file 6: Table S5. Bone volume density (BVD) in percentage (%) of Control and Ca-ion surfaces after 8 weeks of implantation from two ground sections (GS) of each of the 16 implants placed in 8 rabbits. Results are shown as mean ± SD. [file 40729_2021_314_MOESM6_ESM.docx]

| 8 weeks | Control | | | Ca-ion | | |
| --- | --- | --- | --- | --- | --- | --- |
| BVD | GS 1 | GS 2 | Mean | GS 1 | GS 2 | Mean |
| 10 | 29.03% | 31.14% | 30.09% | 29.62% | 31.31% | 30.47% |
| 11 | 19.68% | 21.62% | 20.65% | 32.43% | 34.08% | 33.25% |
| 12 | 34.98% | 34.64% | 34.81% | 24.95% | 28.60% | 26.77% |
| 14 | 15.75% | 25.59% | 20.67% | 37.89% | 40.49% | 39.19% |
| 15 | 26.37% | 23.96% | 25.17% | 24.17% | 27.40% | 25.78% |
| 16 | 28.84% | 30.87% | 29.85% | 40.22% | 28.79% | 34.51% |
| 17 | 26.68% | 26.12% | 26.40% | 34.02% | 28.59% | 31.31% |
| 18 | 39.84% | 37.43% | 38.63% | 33.76% | 35.96% | 34.86% |
| ~~13~~ |  |  |  |  |  |  |
| Mean | 27.64% | 28.92% | 28.28% | 32.13% | 31.90% | 32.02% |
| SD | 7.68% | 5.49% | 6.48% | 5.69% | 4.59% | 4.99% |

Table S 5 Bone volume density (BVD) in percentage (%) of Control and Ca-ion surfaces after 8 weeks of implantation from two ground sections (GS) of each of the 16 implants placed in 8 rabbits. Results are shown as mean ± SD.
